# Current etiology of hypertension in European children — factors associated with primary hypertension

**DOI:** 10.1007/s00467-025-06761-x

**Published:** 2025-05-20

**Authors:** Łukasz Obrycki, Krzysztof Skoczyński, Maksymilian Sikorski, Jan Koziej, Kacper Mitoraj, Jakub Pilip, Michał Pac, Janusz Feber, Mieczysław Litwin

**Affiliations:** 1 Department of Nephrology, Kidney Transplantation and Hypertension, Children’s Memorial Health Institute, Aleja Dzieci Polskich 20, 04 - 730 Warsaw, Poland; 2 Faculty of Medicine, Collegium Medicum, Cardinal Stefan Wyszyński University, Warsaw, Poland; 3 Division of Nephrology, Department of Pediatrics, The Children’s Hospital of Eastern Ontario, Ottawa, Canada; 4 Department of Pediatrics, University Hospital Ostrava and Faculty of Medicine, University of Ostrava, Ostrava, Czech Republic

**Keywords:** Pediatric hypertension, Primary hypertension, Secondary hypertension, Serum uric acid, Hypertension etiology

## Abstract

**Background:**

While hypertension (HT) in pediatric patients is often secondary (SH), recent trends show a rise in primary hypertension (PH), which is associated with an increasing global prevalence of obesity. A relationship between serum uric acid and PH has also been suggested. Our study aimed to assess the etiology of HT and factors associated with PH in a large European cohort of children referred for HT based on office blood pressure (BP) measurements.

**Methods:**

We performed a retrospective analysis of 2008 children aged 0–18 years (12.3 ± 4.9 years) diagnosed with HT. Patients were classified into white coat hypertension (WCH), PH, or SH groups based on office BP, 24-h ambulatory BP monitoring (ABPM) and clinical evaluation. Anthropometric, hemodynamic, and biochemical data were collected.

**Results:**

Out of 2008 patients included in the analysis, 200 (10%) were excluded due to multifactorial HT diagnosis after kidney transplantation (KTx). Among the remaining patients HT was confirmed in 1260 (548 were classified as WCH). Of 1260 patients with HT: 49.3% had PH, while 50.7% SH, mainly secondary to renal parenchymal disease (43.5% of SH patients), aortic coarctation (20.7%), and renovascular HT (18%). Age > 12.5 years, obesity (*BMI SDS* (standard deviation score) ≥ 1.65), and serum uric acid > 4.8 mg/dL were identified as significant factors associated with PH.

**Conclusions:**

Our study provides valuable insights into the current etiology of pediatric HT and highlights the role of age, obesity, and uric acid level in the diagnosis of PH in children.

**Graphical abstract:**

**Figure:**
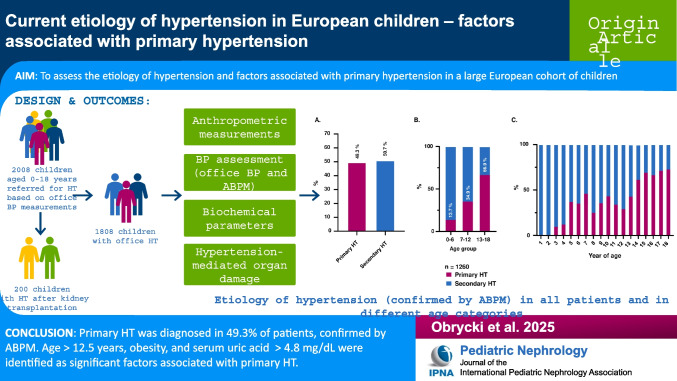
A higher resolution version of the Graphical abstract is available as Supplementary information

**Supplementary Information:**

The online version contains supplementary material available at 10.1007/s00467-025-06761-x.

## Introduction

Hypertension (HT), one of the main, potentially modifiable cardiovascular risk factors, is a significant clinical problem not only in adult patients but also in children and adolescents. Based on a recent systematic review and meta-analysis by Song et al., it can be estimated that HT affects 4–6% of the pediatric population aged 0–18 years, with varying frequencies in individual age groups [1]. In addition to its primary form (primary hypertension, PH), HT can be caused by other chronic diseases such as diabetes, chronic kidney disease (CKD), vascular pathologies, e.g., coarctation of the aorta, renovascular hypertension, mid-aortic syndrome, and others [2–9]. However, detailed data on the etiology of HT in the pediatric population is lacking.

Most studies generally show that PH is less common than secondary hypertension (SH) [2–5] in this age group, but the proportion of PH in pediatric HT patients varied across studies from 15% [2] to even 46% [5]. Considering an increasing proportion of patients with PH, it is important to distinguish PH from SH at an early stage of the work-up. Data published so far show that at the time of diagnosis, patients with PH are older, overweight, and more often have a family history of hypertension than SH patients [2, 6]. Studies conducted in recent years have shown that patients with PH also have higher serum uric acid concentrations, which may suggest the role of this parameter in the diagnosis of PH [10, 11].

The aim of our study was to evaluate the etiology of hypertension and factors associated with PH. We hypothesized that primary hypertension would be associated with obesity and increased serum uric acid level.

## Methods

### Study population

An electronic database was used to identify all patients with confirmed or suspected diagnosis of HT. The initial database query included 2053 patients, 45 of whom were subsequently excluded from the analysis due to lack of data. A total of 2008 children 0–18 years old were initially included (752 girls; mean age 12.3 ± 4.9 years) who had been diagnosed with HT before referral or referred to Children’s Memorial Health Institute in Warsaw (reference hospital for children in Poland with all types of kidney diseases and hypertension) to confirm the diagnosis of HT between January 2012 and December 2022. Patients were referred to our center by primary care physicians or other departments of our hospital, such as neurology, endocrinology, and cardiology. A total of 200 patients after kidney transplantation were excluded, as those patients are not eligible to be diagnosed as having PH, and this would result in a biased analysis of prevalence of PH and SH. Our study is a retrospective study, approved by the Bioethics Committee of the Children’s Memorial Health Institute (IRB number 13/KBE/2023), and was performed in accordance with the Declaration of Helsinki 1975, revised in 2013.

### Data collection

Patients identified in the electronic database were retrospectively manually reviewed. The date of the initial diagnosis of HT or first attendance in the HT clinic/inpatient ward was identified. Then, patient clinical characteristics were collected: age, biological sex, anthropometric measurements (weight, height, body mass index (BMI), and waist circumference), hypertension history, concomitant diseases, laboratory tests (serum creatinine with the calculation of glomerular filtration rate (GFR), serum uric acid level, lipid profile, plasma glucose level, albumin excretion), blood pressure (BP) measurements (office BP, 24-h ambulatory BP monitoring (ABPM)), echocardiography measurements (including left ventricular mass index (LVMi)), carotid intima-media thickness (cIMT), pulse wave velocity (PWV) and pulse wave analysis (PWA) including central systolic blood pressure (cSBP). All the above mentioned examinations and tests are standard in our center and are performed in all hospitalized patients with suspected hypertension (except for the youngest patients, usually under 5 years of age, in whom it is not possible for technical reasons).

### Anthropometrics

Height was measured in the upright standing position in older children; in children whose standing height cannot be measured (usually younger than 2 years), height was measured using the portable Harpenden Infantometer and recorded to the nearest 0.5 cm. Body weight was recorded with an accuracy of 0.1 kg using a digital medical scale in participants who wore light underwear. Body mass index (*BMI*) was calculated according to the formula: *BMI* = body weight (kg)/(height [m])^2^ and expressed in absolute and standard deviation score (*SDS*) values based on Polish national normative data [12, 13]. Waist circumference (*WC*) was measured midway between the lowest rib and the superior border of the iliac crest at the end of a normal expiration with a flexible nonelastic anthropometric tape, to the nearest 0.1 cm and expressed in absolute and *SDS* values based on Polish national normative data [14].

### Hypertension diagnosis

Patient office systolic (SBP) and diastolic (DBP) blood pressure values were measured using validated oscillometric devices at least three times during three independent measurements, from which the mean value was calculated. Patients were classified into hypertension stages according to the European Society of Hypertension and Polish Society of Hypertension recommendations [15, 16], using American reference values (4th Task Force report) for children < 2.5 years old, Polish reference normative values for patients between 2.5 and 16 years old and with standardized cut-off values for older patients [15–19]. Office BP was expressed in absolute and *SDS* values based on the appropriate normative data [17, 18] for the whole age range to allow comparison between groups.

Twenty-four-hour ABPM measurements were performed according to our center protocol during hospital stay using an oscillometric device (SpaceLabs Monitor 90207) with the most appropriate size of cuff fitted to the non-dominant arm. Out of 1808 patients ABPM was not performed in 199 children < 5 years old (in this group, HT was confirmed by repeated office measurements). Readings of SBP, DBP, mean arterial pressure (MAP), pulse pressure (PP), and heart rate (HR) were taken every 20 min during the daytime and every 30 min at night. ABPM was considered valid if ≥ 80% of readings were successful. Patients were instructed to complete a diary recording the type and duration of physical activity and sleep period to define daytime/nighttime periods. Twenty-four-hour BP was expressed in absolute values and in *SDS* values based on the appropriate normative data [20]. Based on collected data, patients were classified into one of the four groups according to recent ESH guidelines (normotension, ambulatory prehypertension, ambulatory hypertension, and severe ambulatory hypertension) [15].

### Echocardiography

All echocardiography examinations were performed by examiners in the Echocardiography Laboratory of the Children’s Memorial Health Institute, according to the European Association of Cardiovascular Imaging and American Society of Echocardiography guidelines [21, 22]. The left ventricular mass index (LVMi) was calculated according to de Simone’s formula as a ratio between left ventricular mass and the patient’s height (in meters) to the power of 2.7. To compare results in patients in all age groups, left ventricular hypertrophy (LVH) was defined as an LVMi value ≥ 95 th percentile for age- and sex-based reference data [23].

### Carotid intima-media thickness and wall cross-sectional area

The cIMT measurements were performed in patients ≥ 6 years old according to the Mannheim Consensus recommendations using the Aloca Prosound Alpha- 7 ultrasound device with a 5.5 to 12.5 MHz linear probe [24]. The results were given as the mean value of the measurements performed in the left and right common carotid arteries. For analysis of patients in different age groups, both absolute and *SDS* values (obtained by the LMS method) were included. *cIMT SDS* ≥ 1.65 (≥ 95 percentile) was considered as a cut-off point for increased cIMT (a marker of hypertensive vascular injury) [25–27]. Additionally, the wall cross-sectional area (*WCSA*) of the common carotid artery was calculated with the use of the following equation:

$$WCSA=\uppi\;{(dD/2+cIMT)}^{2}-\uppi\;{(dD/2)}^{2}$$

where *dD* is the mean diastolic diameter of the artery.

### Pulse wave velocity and pulse wave analysis

Data from the oscillometric Vicorder (SMT Medical®) system device were collected, including carotid-femoral pulse wave velocity (PWV), and central systolic blood pressure (cSBP). Measurements were performed according to the published guidelines in the supine position after 5 min of rest, at the same time as the BP measurement. Both for PWV and pulse wave analysis (PWA), the first few waves were omitted and, when at least 5 next pulse waves were of good quality, 10–15 consecutive pulse waves (heartbeats) were taken for analysis [28, 29].

The PWV in m/s (absolute values calculated by the device) were subsequently converted to *SDS* values based on pediatric normative data [28–30]. PWV *SDS* ≥ 1.88 (≥ 97th percentile) were considered as a cut-off point for the increased PWV (a marker of functional hypertensive injury) [28].

*cSBP* values were interpreted using pediatric normative values obtained with an oscillometric device and *cSBP* ≥ 95th percentile was considered increased [31].

### Laboratory investigations

Specific biochemical blood serum parameters such as total cholesterol (TC), high-density lipoprotein (HDL), low-density lipoprotein (LDL), triglycerides (TG), glucose, insulin, and uric acid (UA) were assessed at the time of diagnosis. In all patients > 6 years old and, when possible, in younger ones, blood samples were taken after 12 h of fasting. Additionally, the data on 24-h urinary albumin excretion was collected. Kidney function was assessed using the serum creatinine level measured by the enzymatic method with commercial kits using an autoanalyzer A15 (BioSystems). The estimated glomerular filtration rate (GFR) was calculated with the modified bedside Schwartz formula [32]:

$$GFR\;[\text{mL}/\text{min}/1.73{\text{m}}^{2}]=0.413\times \text{H }[\text{cm}]/SCr\;[\text{mg}/\text{dL}]$$

*H* — body length/height and *SCr* — serum creatinine level. Normal *GFR* was defined as a *GFR* value ≥ 90 mL/min/1.73 m^2^.

### Classification of patients

All study patients were classified into three groups: white coat hypertension (WCH), primary hypertension, and secondary hypertension. Criteria for the diagnosis of WCH were (i) hypertension diagnosed based on office BP (all participants), (ii) normotension or ambulatory prehypertension in ABPM measurements, and (iii) no pharmacological antihypertensive treatment.

Patients were diagnosed with primary or secondary hypertension by the attending physician based on available information from the work-up. Initial work-up, including laboratory tests, echocardiography and abdominal ultrasound was the same for all patients. More advanced diagnostics, such as computed tomography (CT), magnetic resonance imaging (MRI), kidney scintigraphy, and genetic tests were performed based on clinical indications as deemed appropriate by the attending physician.

Patients were classified as primary hypertension if (i) WCH was ruled out, (ii) no secondary causes of hypertension were found, and (iii) no medications with the potential to raise BP were administered.

Patients were diagnosed with secondary hypertension (SH) after extensive evaluation of all available data and identification of the cause of hypertension. Those patients were further classified into 12 groups based on SH etiology: 1 — renovascular, 2 — middle-aortic syndrome (MAS), 3 — coarctation of the aorta (CoA), 4 — renal parenchymal, 5 — pheochromocytoma/paraganglioma, 6 — monogenic, 7 — central nervous system (CNS) disorders, 8 — polycystic ovary syndrome (PCOS), 9 — drug-induced, 10 — adrenal diseases, 11 — neuroblastoma, 12 — perinatal history (including prematurity).

### Statistical analyses

All analyzed parameters were checked for normality of distribution with the Shapiro–Wilk test. Because some variables were non-normally distributed, all variables are shown as medians with interquartile ranges and compared (univariate analysis) using a nonparametric Kruskal–Wallis test. Statistically significant differences/parameters between PH and SH groups on univariate analysis were included as predictors in stepwise backward elimination multivariate regression with primary hypertension as the dependent/outcome variable. The final set of predictors/independent variables was further analyzed with receiver operator curve (*ROC*) to obtain sensitivity, specificity, area under the curve (*AUC*) and equal error rate (*EER*)/best threshold value for the association with *PH* as outcome variable.

Multivariate logistic regression was then performed with *PH* as the dependent variable and predictors classified into binary variables based on the optimal cut-off values obtained from the *ROC* analysis. The multivariate regression and logistic regression analysis was performed in all patients and separately for patients with normal *GFR*. *P* value of < 0.05 was considered statistically significant.

## Results

### Classification of patients

**Fig. 1:**
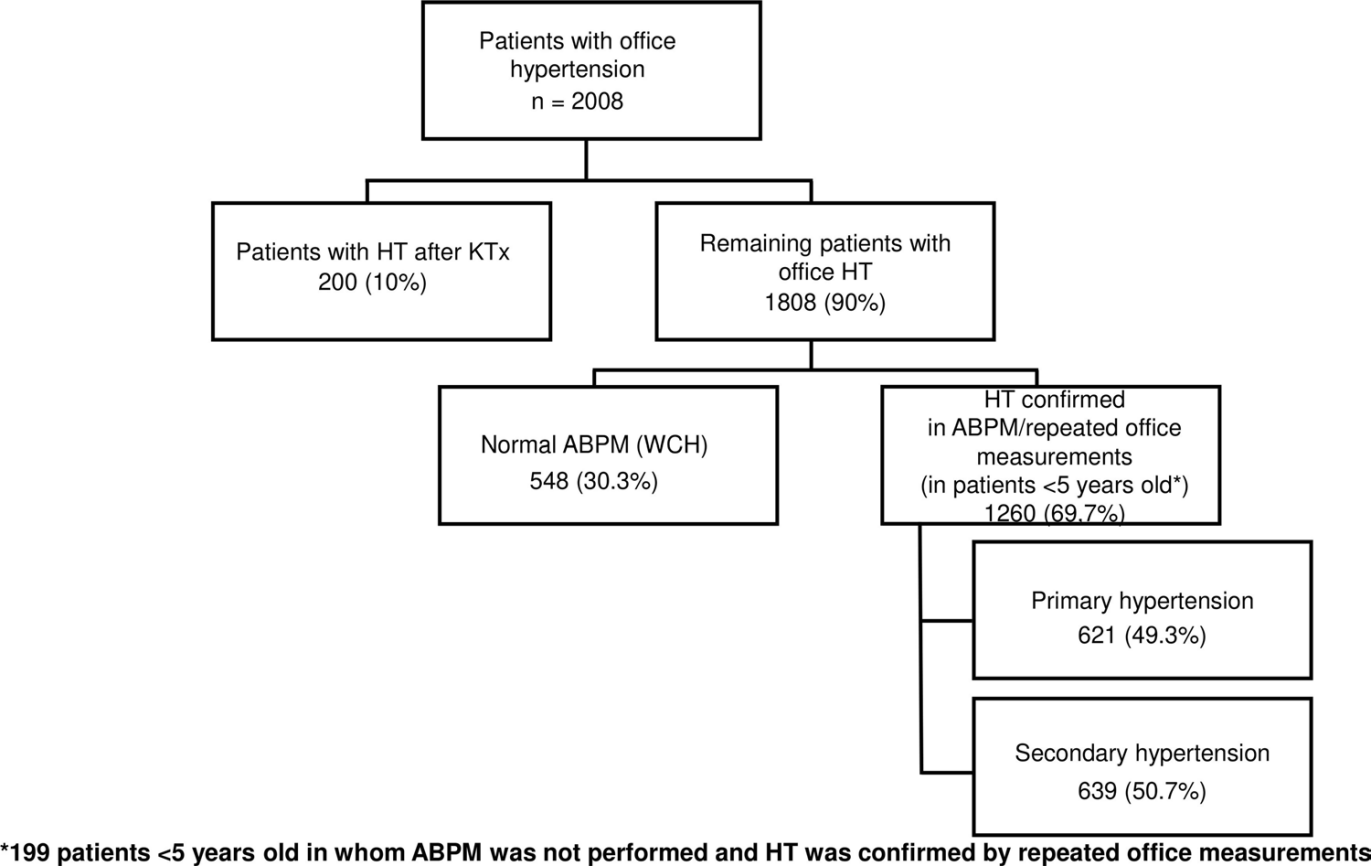
Scheme of the study

Out of 2008 patients (752 girls; 12.3 ± 4.9 years old), after excluding kidney transplant patients, ABPM was performed in patients ≥ 5 years old which revealed WCH in 548 subjects (185 girls; 13.7 ± 3.9 years old), and this group was subsequently excluded from further analysis (Fig. 1). HT was confirmed in 1260 patients (484 girls; 11.6 ± 4.9 years old), and this group was included in the final analysis. The number of patients with confirmed HT increased with patient age, with a significant increase from the 14th year of age (Supplementary Fig. S1).

**Fig. 2:**
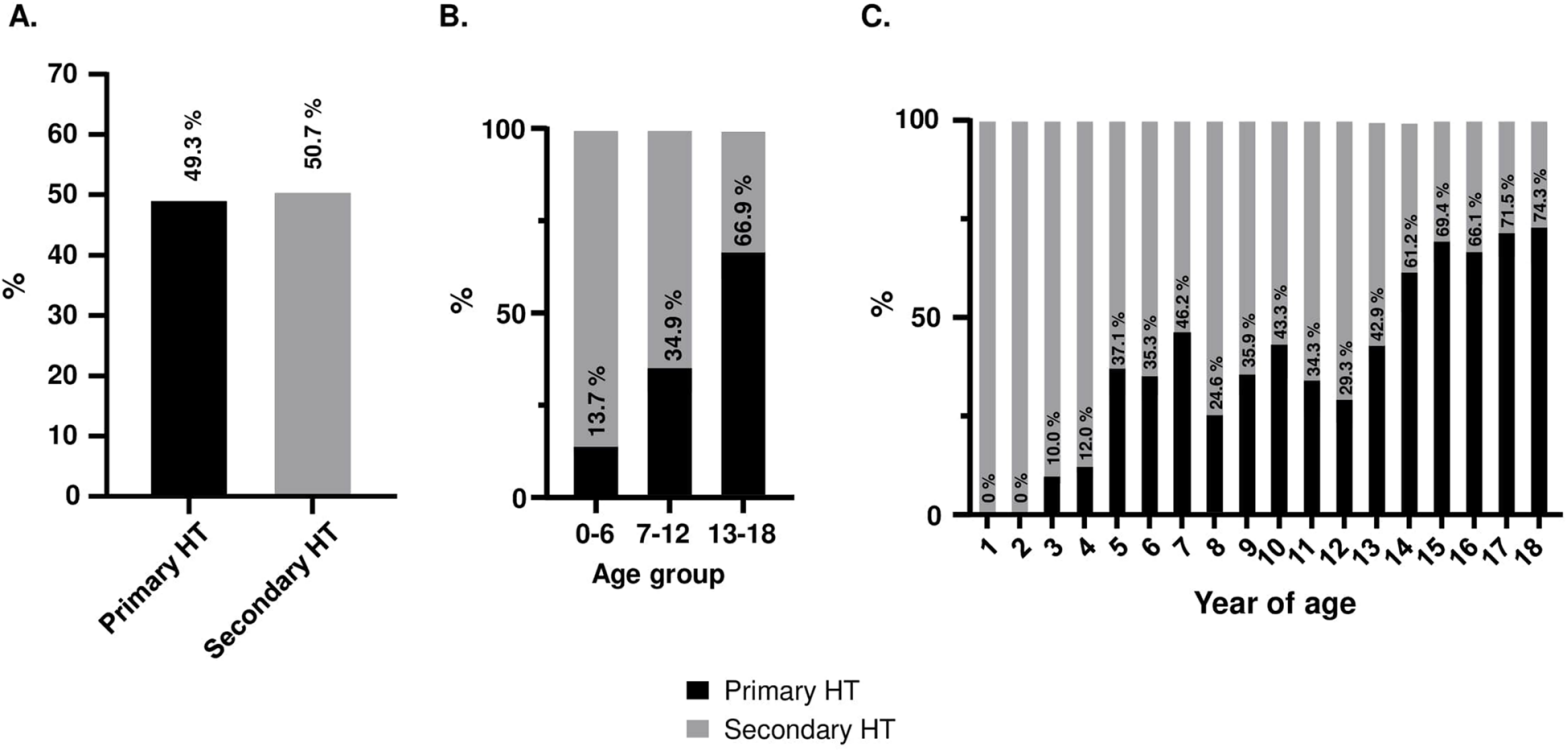
Etiology of hypertension in all patients (**A**) and in different age groups (**B-C**)

After extensive evaluation of all patients, PH was diagnosed in 621 patients (49.3%) and SH in 639 (50.7%) patients (Fig. 2A). After dividing the patients into age categories, the proportion of patients with PH increased from 33 patients (13.7%) in the group 0–6 years of age; through 105 (34.9%) aged 7–12 years up to 483 (66.9%) patients in the group 13–18 years of age (Fig. 2B-C). To exclude the effect of obesity and overweight on the prevalence of PH and SH among HT patients, we performed an additional analysis in normal weight subjects of whom 40.7% were diagnosed with PH and 59.3% with SH.

### Secondary causes of hypertension

**Fig. 3:**
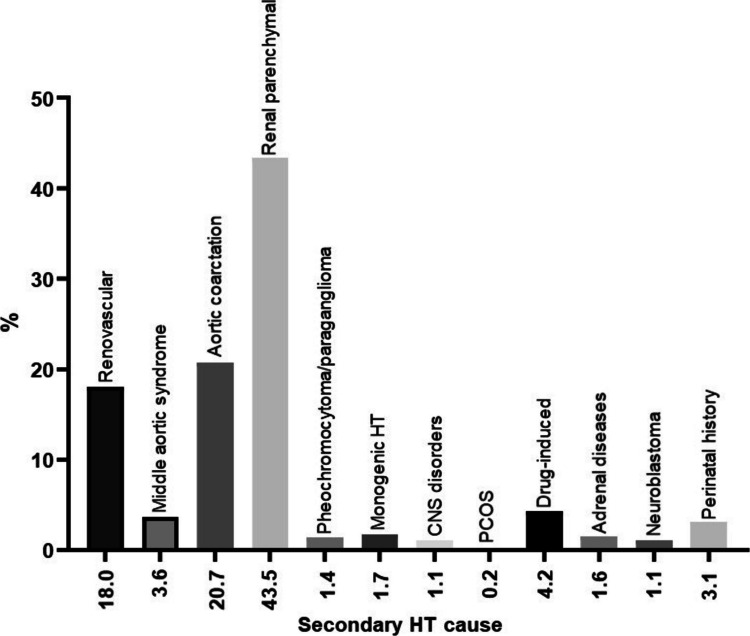
Detailed etiology of hypertension in patients with secondary hypertension. HTN, hypertension; KTx, kidney transplantation; CNS, central nervous system; PCOS, polycystic ovary syndrome

Among patients diagnosed with SH, the most common causes were as follows: renal parenchymal disease in 276 patients (43.5%), coarctation of the aorta (CoA) in 132 (20.7%), and renovascular HT in 115 (18%) subjects (Fig. 3). Those three causes were most frequent in all age groups. The only change in their distribution was observed in the group 0–6 years of age, where renovascular cause (17.4%) emerged as the second most common after renal parenchymal etiology (43.3%).

Other common conditions responsible for SH were middle-aortic syndrome (MAS) in 23 patients (3.6%), drug-induced hypertension in 27 (4.2%), and perinatal history (including prematurity) in 20 (3.1%). The rest of the causes were observed in 0.2–1.7% of the patients (Fig. 3).

### Comparison of patients with PH and SH

There were notable anthropometric, hemodynamic, and biochemical differences between patients with PH and SH (Table 1). Patients with PH were older (median age 15.1 vs. 9.6 years, *p* < 0.001). However, PH was diagnosed as early as 3 years of age in 4 children (10%). All measured anthropometric variables were higher in the PH group; of particular note are measurements related to the risk of overweight/obesity and metabolic syndrome — *BMI SDS* (1.04 vs. 0.25; *p* < 0.001) and *WC SDS* (1.23 vs. 0.45, *p* < 0.001). Consequently, there are significantly more patients with overweight (279 patients (45%) vs. 202 (32%); *p* < 0.001) and obesity (230 (37%) vs. 38 (6%); *p* < 0.001) in the PH group. There were no significant differences in perinatal parameters (birth weight, Apgar score) between groups.

**Table 1:** Comparison of patients with primary and secondary hypertension

Variable	Primary hypertension (*n* = 621)	Secondary hypertension n = (639)	*p* value
Sex (%)	♀212 (34.1)	♀272 (42.6)	0.002
	♂409 (65.9)	♂367 (57.4)	
Age [years]	15.1 [12.7–16.8]	9.6 [4.1–14.2]	< 0.001
Weight [kg]	70.5 [55.0–84.0]	34.0 [17.2–55.4]	< 0.001
Height [cm]	169.0 [158.0–178.0]	142.2 [110.1–162.0]	< 0.001
*BMI* [kg/m^2^]	24.0 [21.1–28.3]	18.1 [15.5–21.4]	< 0.001
*BMI-SDS*	1.04 [0.16–1.88]	0.25 [− 0.81–1.23]	< 0.001
*BMI-SDS* ≥ 1.04* [no. of patients; (%)]	279 [45]	202 [32]	< 0.001
*BMI-SDS* ≥ 1.65† [no. of patients; (%)]	230 [37]	38 [6]	< 0.001
*WC* [cm]	79.5 [71.9–88.0]	67.0 [60.5–75.5]	< 0.001
*WC-SDS*	1.23 [0.48–2.05]	0.45 [− 0.45–1.08]	< 0.001
*BW* [kg]	3.28 [2.83–3.65]	3.24 [2.78–3.56]	n.s
Gest age [weeks]	39 [37–40]	39 [37–40]	n.s
Apgar score	10 [9, 10]	10 [9, 10]	n.s
*SBP* [mmHg]	139 [128–150]	129 [117–140]	< 0.001
*SBP-SDS*	2.32 [1.55–2.92]	1.51 [0.11–3.00]	0.02
*DBP* [mmHg]	77 [70–85]	75 [67–85]	n.s
*DBP-SDS*	1.68 [0.84–2.60]	1.59 [0.32–2.95]	n.s
24-h *SBP* [mmHg]	127 [121–134]	127 [121–134]	n.s
24-h *SBP-SDS*	1.86 [0.82–2.45]	2.24 [0.93–3.15]	0.008
24-h *DBP* [mmHg]	73 [67–77]	70 [64–80]	n.s
24-h *DBP-SDS*	0.98 [0.10–1.63]	0.67 [− 0.47–2.12]	n.s
24-h *HR* [‘/min]	79 [71–89]	81 [74–90]	0.04
*cSBP* [mmHg]	119 [112–126]	117 [106–130]	n.s
*HR* [‘/min]	75 [67–83]	88 [78–108]	< 0.001
*PWV* [m/s]	5.8 [5.3–6.3]	5.3 [4.7–6.5]	n.s
*PWV-SDS*	1.8 [0.9–2.7]	1.76 [0.55–2.96]	n.s
*PWV-SDS* ≥ 1.88 [no. of patients; (%)]	188 (47.8)	224 (48.5)	n.s
*WCSA* [mm^2^]	7.1 [6.6–7.9]	7.3 [6.5–8.2]	n.s
*cIMT* [mm]	0.43 [0.41–0.46]	0.44 [0.41–0.49]	0.002
*cIMT-SDS*	0.9 [0.4–1.5]	1.4 [0.63–2.33]	< 0.001
*cIMT-SDS* ≥ 1.65 (no. of patients; (%))	78 (19.8)	187 (40.4)	0.001
LVMi [g/m height^2.7^]	35.1 [30.4–40.2]	37.4 [31.6–43.9]	< 0.001
LVH [no. of patients; (%)]	93 (16.9)	159 [25.9]	0.009
Creatinine [mg/dL]	0.72 [0.59–0.86]	0.63 [0.44–0.91]	< 0.001
*GFR* [mL/min/1.73 m^2^]	94.2 [83.5–109.4]	92.5 [72.1–114.2]	0.04
Uric acid [mg/dL]	5.6 [4.8–6.6]	4.7 [3.8–6.0]	< 0.001
Glucose [mg/dL]	86 [81–91]	87 [81–95]	0.029
Insulin [IU/mL]	13.9 [10.6–19.2]	12.6 [6.9–18.2]	n.s
Total cholesterol [mg/dL]	159 [137–182]	166 [144–191]	0.001
LDL cholesterol [mg/dL]	87 [72–107]	90 [72–111]	n.s
HDL cholesterol [mg/dL]	50 [42–60]	54 [45–64]	< 0.001
Triglicerydes [mg/dL]	85 [65–123]	84 [62–135]	n.s
MALB [mg/24 h]	8.1 [4.7–14.7]	8.8 [4.8–24.6]	n.s

*BMI* body mass index, *WC* waist circumference, *BW* birth weight, *Gest age* gestational age, *SBP* systolic blood pressure, *DBP* diastolic blood pressure, *24-h SBP* 24-h systolic blood pressure, *24-h DBP* 24-h diastolic blood pressure, *24-h HR* 24-h heart rate, *cSBP* central systolic blood pressure, *HR* heart rate, *PWV* pulse wave velocity, *PWV-SDS* pulse wave velocity standard deviation score, *WCSA* wall cross-sectional area, *cIMT* carotid intima-media thickness, *cIMT-SDS* carotid intima-media thickness standard deviation score, *LVMI* left ventricular mass index, *LVM/BSA* left ventricular mass/body surface area, *LVH* left ventricular hypertrophy, *GFR* glomerular filtration rate, *HDL* high-density lipoprotein, *LDL* low-density lipoprotein, *MALB* microalbumin excretion

^*^ *BMI-SDS* ≥ 1.04 corresponds to the 85th percentile (cut-off value for overweight)

^†^*BMI-SDS* ≥ 1.65 corresponds to the 95th percentile (cut-off value for obesity)

Although office DBP *SDS* values did not differ between the PH and SH groups, the PH group showed higher office SBP *SDS* values (2.32 vs. 1.51, *p* = 0.02). In ABPM, we also found significant differences in 24-h SBP *SDS* values (1.86 vs. 2.24, *p* = 0.008) between PH and SH patients.

Consistently, patients with SH presented signs of hypertension-mediated organ damage (HMOD) more frequently: LVMi was higher (37.4 vs. 35.1 g/m^2,7^; *p* < 0.001) and LVH was more frequent (25.9% vs. 16.9%; *p* = 0.009) in the SH group in comparison with the PH group. Patients with SH were also more prone to present signs of vascular damage resulting in higher cIMT (0.44 vs. 0.43 mm, *p* = 0.002) and cIMT *SDS* values (1.4 vs. 0.9, *p* < 0.001). There were no significant differences between groups in PWV, in both absolute and *SDS* values.

Significant differences were also found between groups in biochemical parameters (Table 1). The PH group had higher *GFR* (94.2 vs. 92.5 mL/min/1.73 m^2^; *p* = 0.04), higher serum uric acid (5.6 vs. 4.7 mg/dL; *p* < 0.001), and lower glucose levels (86 vs. 87 mg/dL; *p* = 0.029). A comparison of patients with PH and SH and those with normal kidney function (*GFR* ≥ 90 mL/min/1.73 m^2^) showed even greater differences in serum uric acid concentrations (5.6 vs. 4.4 mg/dL; *p* < 0.001). Overall, 91 (56%) of patients with PH had uric acid levels above 5.5 mg/dL compared to 13 (15.7%) patients with SH (after excluding those with *GFR* < 90 mL/min/1.73 m^2^).

Interestingly, patients with PH had significantly different lipid profiles: lower total cholesterol (159 vs. 166 mg/dL; *p* = 0.001), not significantly lower LDL cholesterol (87 vs. 90 mg/dL; n.s.), but significantly lower HDL cholesterol (50 vs. 54 mg/dL; *p* < 0.001).

### Factors associated with primary hypertension

Out of all significantly different demographic and biochemical parameters (between PH and SH) identified on univariate analysis (Table 1) the following parameters were included in the final model of multivariate analysis: age, *BMI SDS* and serum uric acid level. *GFR* and *GFR* ≥ 90 mL/min/1.73 m^2^ were included in the multivariate analysis of the whole group but excluded from the analysis of patients with normal *GFR*.

We did not include weight, height, *BMI* and *WC* as these parameters (in absolute values) are not comparable among children of different age; *WC SDS* was excluded due to cross-correlation with *BMI SDS*. We also did not include lipid profile parameters due to significant variation of these parameters across pediatric age and the fact that some children had the lipid profile done in a non-fasting state.

**Fig. 4:**
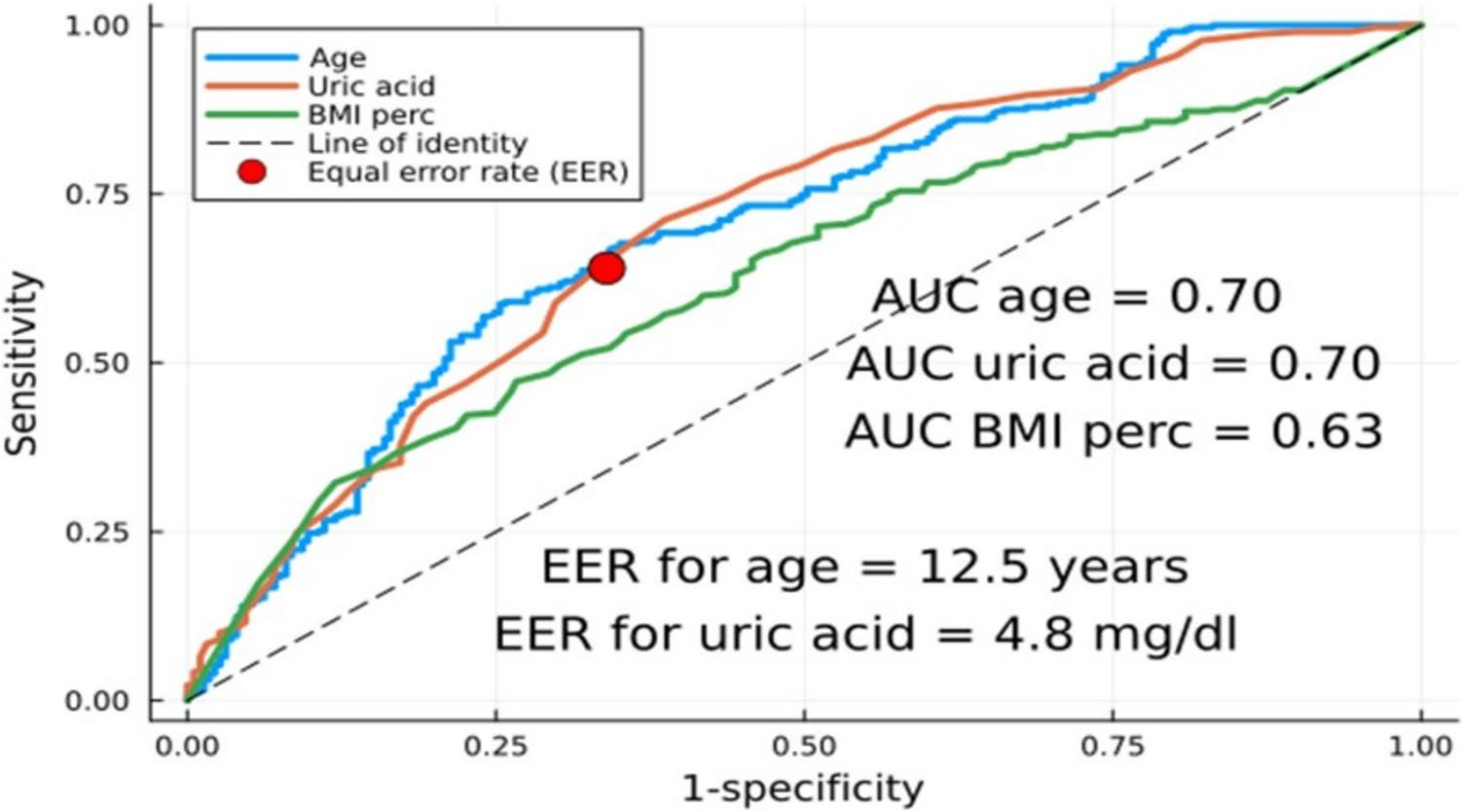
Sensitivity and specificity of age, *BMI* percentile, and serum uric acid level as factors associated with primary hypertension in patients with normal *GFR*

*ROC* analysis and multivariate logistic regression (with cut-off points obtained by *ROC* analysis) confirmed that age > 12.5 years, *BMI* ≥ 95 th percentile, *GFR* ≥ 90 ml/min/1.73 m^2^ and serum uric acid level > 4.8 mg/dL are significantly associated with PH (Fig. 4; Table 2) . After exclusion of patients with impaired kidney function (*GFR* < 90 mL/min/1.73 m^2^), the most significant factors associated with PH were uric acid > 4.8 mg/dL with an odds ratio (*OR*) of 1.8 (95% *CI* = 1.4–2.32), followed by *BMI* ≥ 95 percentile (*OR* 1.68, 95% *CI* = 1.27–2.25) and age > 12.5 years (*OR* = 1.6, 95% *CI* = 1.26–2.05) (Fig. 4; Table 3). To exclude the effect of obesity and overweight on the calculation of factors associated with PH, we performed an additional analysis excluding overweight and obese patients. Serum uric acid and age remained significant factors associated with PH (Supplementary Table S1).

**Table 2:** Significant factors associated with primary hypertension in all patients

Characteristic	*β*	*β* 95% *CI*	*p* value	*OR*	*OR* 95% *CI*
Multivariate regression with continuous variables					
Age	0.11	0.09–0.13	< 0.001	1.12	1.09–1.14
*BMI-SDS*	0.006	0.003–0.009	< 0.001	1.006	1.003–1.009
Uric acid	0.19	0.12–0.26	< 0.001	1.21	1.12–1.30
*GFR*	0.01	0.01–0.02	< 0.001	1.01	1.01–1.02
Multivariate logistic regression					
Age > 12.5 y.o	0.81	0.61–1.01	< 0.001	2.25	1.84–2.75
*BMI-SDS* ≥ 1.65	0.40	0.18–0.62	< 0.001	1.49	1.20–1.86
Uric acid > 4.8 mg/dL	0.57	0.36–0.77	< 0.001	1.77	1.43–2.16
*GFR* ≥ 90 mL/min/1.73 m^2^	0.47	0.27–0.66	< 0.001	1.60	1.31–1.93

*BMI* body mass index, *CI* confidence interval, *GFR* glomerular filtration rate, *OR* odds ratio

**Table 3:** Significant factors associated with primary hypertension factors in patients with normal *GFR*

Characteristic	*β*	*β* 95% CI	*p* value	*OR*	*OR* 95% *CI*
Multivariate regression with continuous variables					
Age	0.06	0.03–0.09	< 0.001	1.06	1.03–1.09
*BMI-SDS*	0.004	0.001–0.008	0.008	1.004	1.00–1.01
Uric acid	0.24	0.14–0.34	< 0.001	1.28	1.15–1.40
Multivariate logistic regression					
Age > 12.5 y.o	0.47	0.23–0.72	< 0.001	1.60	1.26–2.05
*BMI-SDS* ≥ 1.65	0.52	0.24–0.81	< 0.001	1.68	1.27–2.25
Uric acid > 4.8 mg/dL	0.59	0.34–0.84	< 0.001	1.80	1.40–2.32

*BMI* body mass index, *CI* confidence interval, *OR* odds ratio

## Discussion

The main outcome of our study is a detailed description of the current (2012–2022) etiology of hypertension in children and adolescents in the largest European cohort of pediatric patients, which is a sample of the referral population to a hypertension center. Another important message is the association between serum uric acid level and PH.

The percentage of patients with PH in our group of patients (0–18 years) was 49.3%. This is higher than in a study by Chen et al. performed in a similar age group (0–17 years). However, in the study by Chen et al., the BP measurement method was not specified, and it is also unclear whether hypertension diagnosed in patients was confirmed by ABPM [4]. In another study conducted in Turkey (Çakici et al.) among 383 participants aged 0–19 years (mean age 13.4 years), the percentage of patients with PH with hypertension confirmed by ABPM was 51.5% [9]. This difference may be due to the younger age of our patients (11.8 years). Our results compare favorably with a study by Gupta-Malhotra et al., who observed a similar proportion of patients with PH (43%) in a group of 275 hypertensive (office and ABPM) children (3–17 years old) [5]. When comparing prevalence of PH patients in different age categories, our results are like the study performed by Flynn et al. in the youngest (0–6 years of age: 16.7% compared to 13.7% in our study) and in the oldest (13–18 years of age) group (60% vs. 66.9%). In contrast, in our study we observed a lower prevalence of PH in the group of 7–12-year-old patients (34.9% vs. 61.8% in the group by Flynn et al. [33]) (Fig. 2B).

The results of our study can also be compared to the study by Wyszyńska et al. from 1992, who analyzed the etiology of hypertension in 1025 children referred to our center between 1982 and 1989. It is worth emphasizing that in this historical analysis, no case of PH was found in children under 14 years of age, despite the use of less accurate diagnostic methods. This comparison indicates a significant evolution in the etiology of HT in children and adolescents over the last 3 decades [34].

### Etiology of secondary hypertension

The most common cause of SH in our study was renal parenchymal HT (43.4%; Fig. 3), consistent with previously published studies [4, 5, 9, 34, 35]. The other most common causes of SH were CoA (20.7%), followed by renovascular HT (18%). This is similar to a study by Çakici et al. [9], reporting slightly lower percentages of 7% and 9.1%, respectively, and by Wyszyńska et al. [34], reporting 2.3% (with a much greater number diagnosed at the cardiology department) and 9.7%, respectively. In studies by Gupta-Malhotra [5] et al. and Chen et al. [4], the most common causes of SH, after renal parenchymal, were respiratory, rheumatic immune, and hematological diseases. This is most likely also due to the specificity of the centers and the associated available patient pool.

### Comparison of patients with PH and SH

Our cohort demonstrated significant differences in anthropometric measurements, hemodynamic parameters, and biochemical profiles between patients with PH and SH (Table 1). Patients with PH were more likely to be male, which is supported by earlier research [4, 5]. They also have significantly higher *BMI* and *BMI SDS* values than patients in the SH group, as already documented in the studies by Chen et al. [4] and Çakıcı et al. [9]. Our patients with PH also have higher *WC* and *WC SDS* values, as equivalent to abdominal obesity which cannot be compared with other studies because it was not assessed.

Contrary to Gupta-Malhotra et al. [5] who indicated a higher prevalence of preterm birth in the SH patient group, we did not find a significant difference between SH and PH groups.

The patterns of *BP* values and HMOD demonstrated significant differences between PH and SH patients. Patients with PH presented significantly higher office *SBP SDS* with no differences in office *DBP SDS* values. Chen et al. [4] also found significantly higher office *SBP* values in the PH group, but in contrast to our study, Chen also noted significantly higher office *DBP* values in the SH group. There were no differences in office *SBP* and *DBP* values between PH and SH groups in the study by Çakıcı et al. [9], and office *BP* was not assessed in the study by Gupta-Malhotra et al. [5].

In contrast to the study by Çakıcı et al. [9] where patients with SH had significantly higher 24-h *SBP SDS* and 24-h *DBP SDS* values, we found significant differences between PH and SH groups only in terms of 24-h *SBP SDS* values. We cannot compare our ABPM results with studies by Chen et al. [4] and Gupta-Malhotra et al. [5] due to the lack of reported ABPM values in these studies.

Our data shows that patients with SH have a higher incidence of HMOD and more severe HMOD, including higher LVMi values and a greater frequency of LVH. Additionally, patients with SH demonstrated higher cIMT and *cIMT SDS* values, indicating a more advanced vascular injury (Table 1). These findings are consistent with data reported by Çakıcı et al. [9], where the prevalence of LVH was substantial in both PH (36%) and SH patients (27%), and the mean LVMi in all hypertensive patients was 45.3 g/m^2.7^ (compared to 35.1 g/m^2.7^ in PH and 37.4 g/m^2.7^ in SH patients in our groups). However, the study by Çakıcı et al. [9] identified no significant differences in LVH prevalence or LVMi values between the PH and SH groups.

We noted differences in lipid profile between PH and SH patients: PH patients had significantly lower total cholesterol and HDL cholesterol levels. Differences in the lipid levels between PH and SH patients in our study can be explained by the younger age of the SH cohort, which likely contributes to higher lipid levels, as cholesterol and triglycerides naturally fluctuate during pediatric growth phases. Additionally, blood samples in the youngest group (mostly with SH) were taken without fasting, which potentially could affect serum lipid and glucose results. Chen et al. also described dyslipidemia (without specifying absolute values) but only in patients with PH, without reflecting on lipid-related factors in patients with SH [4].

Other biochemical parameters also varied between groups: in SH patients, the 24-h microalbumin excretion was higher (but it was not statistically significant), corresponding to findings by Gupta-Malhotra et al. [5]. The SH group also demonstrated higher glucose levels (Table 1).

### Serum uric acid levels

Elevated serum uric acid levels in patients with PH may play a more direct role in hypertension development beyond being a marker of metabolic dysfunction. Epidemiological studies suggest that hyperuricemia is an independent predictor of hypertension, particularly in younger individuals, and is associated with vascular damage, including increased carotid intima-media thickness [36]. Experimental models further indicate a mechanistic link between serum uric acid and BP elevation via endothelial dysfunction and renal microvascular injury, supporting its role as a potential contributor to PH pathophysiology [36].

In our study, PH patients had significantly higher uric acid levels compared to those with SH (Table 1), which agrees with a previous paper by Feig et al. who compared groups with different blood pressure status and with PH and SH [10, 11]. In Feig’s study, serum uric acid > 5.5 mg/dL was found in 89% of subjects with PH compared to 30% with SH, 0% with WCH, and 0% of the control normotensive group. In our study serum uric acid > 5.5 mg/dL was found in 56% of patients with PH and 15.7% of those with SH. However, our *ROC* analysis showed that even lower serum uric acid level (4.8 mg/dL) had the highest sensitivity and specificity for the association with PH.

### Factors associated with PH — multivariate analysis

In a multivariate regression analysis, older age and higher *BMI SDS* emerged as strong predictors/factors associated with PH. *WC SDS* was not found to be significantly associated with PH in our analysis when analyzed simultaneously with *BMI,* most likely due to strong cross-correlation between *BMI* and *WC*. Our findings agree with the conclusions previously described by various authors, who also identified age and *BMI* as key PH indicators, often accompanied by a family history of hypertension [4, 5, 9, 33]. Other significant factors associated with PH were normal *GFR* and elevated serum uric acid (Table 2). Moreover, after excluding patients with abnormal kidney function, we found that uric acid (UA) level > 4.8 mg/dL was the most significant factor associated with PH (Table 3). Our finding is consistent with studies indicating that increased UA may contribute to early cardiovascular changes by inducing endothelial dysfunction and impairing nitric oxide bioavailability, ultimately leading to elevated blood pressure [37]. Higher UA levels are perceived as one of the non-classical components of metabolic syndrome and have been associated with greater cardiovascular risk, even in young populations [37–40].

## Limitations

Although our study provides detailed information on the current etiology of hypertension in children and adolescents and its evolution over the last decades, it has some limitations. First, the study population was heterogeneous and consisted of children referred for hypertension by primary care physicians as well as those hospitalized for various reasons who were diagnosed with office HT during hospitalization. Second, ABPM measurements were performed as part of a diagnostic work-up during admission to the hospital, which could have influenced *BP* values compared to ABPM performed outside the hospital, which may overestimate the prevalence of sustained hypertension due to the presence of unidentified WCH. Third, we have no data on the socioeconomic status of the patients’ families, nor data on family history of HT. Prenatal history is limited to birth weight, gestational week, and Apgar score. We also do not have information on any other perinatal complications. Moreover, because ABPM was performed only in patients ≥ 5 years old, we were unable to exclude WCH in younger children.

## Conclusions

Our study, including the largest European cohort of children with hypertension described in recent years, provides detailed information on the current etiology of hypertension in children and adolescents and its evolution over the last decades. We confirmed the role of anthropometric parameter assessment in diagnosing PH, but we also showed, based on multivariate regression analysis, that older age, obesity and serum uric acid are strongly associated with PH in children and adolescents. Future research could expand on these determinants to develop a robust, risk-based model for pediatric hypertension screening that includes genetic, metabolic, and lifestyle factors. Such a model would enable more personalized treatment strategies, especially in populations experiencing increasing rates of pediatric obesity and metabolic syndrome. Our study also sets the stage for longitudinal studies to assess the long-term impact of early PH management, particularly in reducing adult cardiovascular risks, e.g., by reducing serum acid levels non-pharmacologically and, when needed, with pharmacotherapy.

## Supplementary Information

Below is the link to the electronic supplementary material.

Supplementary materials (DOCX 77 KB)

Graphical abstract (PPTX 122 KB)

## Data Availability

The datasets generated during and/or analyzed during the current study are available from the corresponding author on reasonable request.
