# Supplementary material for: Current etiology of hypertension in European children — factors associated with primary hypertension
Source: Pediatr Nephrol. 2025 May 20;40(10):3233–44. doi: 10.1007/s00467-025-06761-x (PMC12402006; doi:10.1007/s00467-025-06761-x)

**Supplementary materials**

**Supplementary Table S1. Significant factors associated with primary hypertension in patients with normal GFR and after exclusion of obese and overweight patients.**

| **Multivariate regression with continuous variables** | | | | | |
| --- | --- | --- | --- | --- | --- |
| **Characteristic** | **β** | **β 95% CI** | **p value** | **OR** | **OR 95% CI** |
| Age | 0.08 | 0.04-0.12 | <0.001 | 1.08 | 1.04-1.13 |
| Uric acid | 0.20 | 0.07-0.33 | <0.001 | 1.22 | 1.07-1.39 |
| **Multivariate logistic regression** | | | | | |
| Age > 12.5 y.o. | 0.64 | 0.32-0.95 | <0.001 | 1.90 | 1.38-2.59 |
| Uric acid > 4.8 mg/dL | 0.57 | 0.25-0.89 | <0.001 | 1.77 | 1.28-2.43 |

**Supplementary Figure S1. Number of patients with confirmed HT in each age category.**


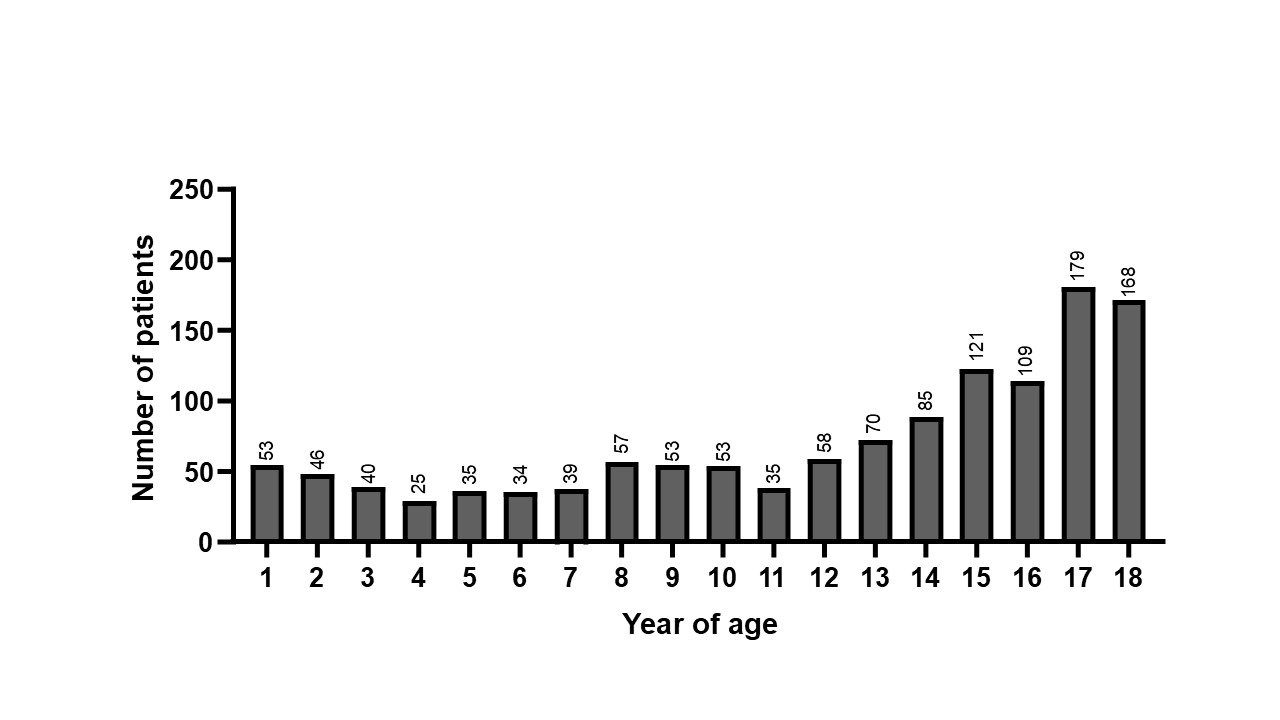

Supplement: Supplementary file 1 — Supplementary materials (DOCX 77 KB) [file 467_2025_6761_MOESM1_ESM.docx]
