# Supplementary material for: Current etiology of hypertension in European children — factors associated with primary hypertension
Source: Pediatr Nephrol. 2025 May 20;40(10):3233–44. doi: 10.1007/s00467-025-06761-x (PMC12402006; doi:10.1007/s00467-025-06761-x)
Supplement: Supplementary file 2 — Graphical abstract (PPTX 122 KB) [file 467_2025_6761_MOESM2_ESM.pptx]

## Slide 1
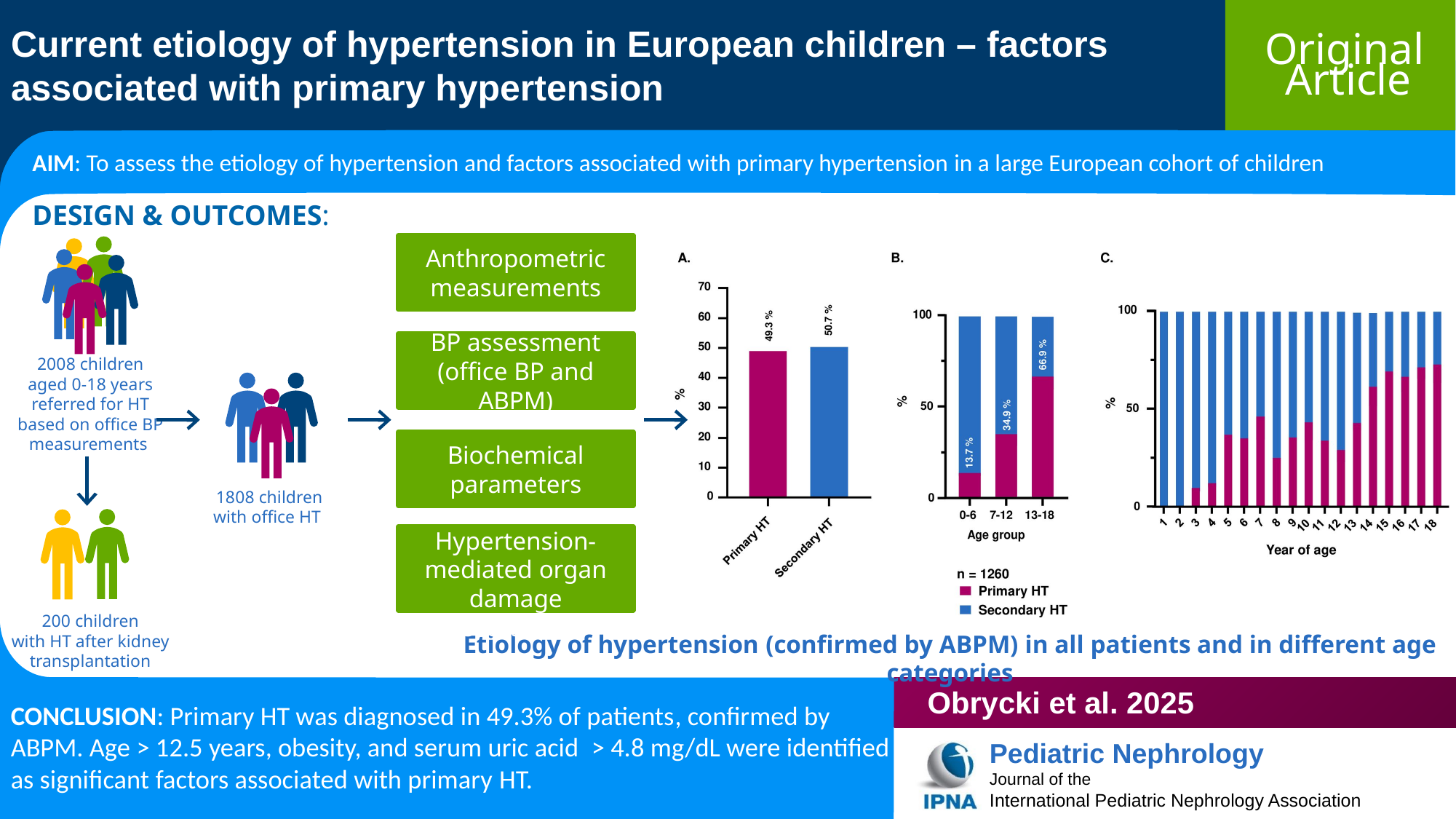

Current etiology of hypertension in European children – factors associated with primary hypertension
AIM: To assess the etiology of hypertension and factors associated with primary hypertension in a large European cohort of children
DESIGN & OUTCOMES:
Anthropometric
measurements
BP assessment (office BP and ABPM)
2008 children
aged 0-18 years
referred for HT based on office BP measurements
1808 children
with office HT
Biochemical parameters
Hypertension-mediated organ damage assessment
200 children
with HT after kidney transplantation
Etiology of hypertension (confirmed by ABPM) in all patients and in different age categories
Obrycki et al. 2025
CONCLUSION: Primary HT was diagnosed in 49.3% of patients, confirmed by ABPM. Age > 12.5 years, obesity, and serum uric acid > 4.8 mg/dL were identified as significant factors associated with primary HT.
